# Cushing's Syndrome Behind Hypokalemia and Severe Infection: A Case Report

**DOI:** 10.7759/cureus.32486

**Published:** 2022-12-13

**Authors:** Catarina Elias, Diana Oliveira, Maria Manuel Silva, Patrícia Lourenço

**Affiliations:** 1 Internal Medicine, Centro Hospitalar Universitário São João, Porto, PRT; 2 Internal Medicine, Centro Hospitalar e Universitário de São João, Porto, PRT; 3 Endocrinology, Diabetes and Metabolism, Centro Hospitalar Universitário de São João, Porto, PRT; 4 Internal Medicine, Centro Hospitalar Universitário de São João, Porto, PRT

**Keywords:** cushing's disease, hypokalemia, infection, hypercortisolism, cushing’s syndrome

## Abstract

Cushing’s syndrome (CS) is a rare condition associated with increased morbidity and mortality. Complications derive from hypercortisolism and are mainly cardiovascular, infectious and thrombotic. Most manifestations are unspecific, and the diagnosis is frequently delayed and made only in the setting of complications.

We present a woman in whom CS was investigated because of refractory hypokalemia, hypernatremia and metabolic alkalosis. The patient had many cardiovascular risk factors and was admitted to the hospital due to a serious bacterial infection - muscle abscesses evolving into osteomyelitis. The final etiological diagnosis was not possible because the acute event had a fatal outcome.

Immunosuppression associated with hypercortisolism makes these patients predisposed to severe infection. Indeed, infectious complications are a relevant cause of death in CS. Diagnosing and treating CS early is paramount in preventing its dismal complications.

## Introduction

Cushing's syndrome (CS) is a rare entity with elevated morbidity and mortality, particularly if not timely diagnosed and treated [1,2]. Cardiovascular disease, thrombotic events and infections are common complications [3,4]. Manifestations of hypercortisolism are widely unspecific and common and this accounts for much of the diagnostic delay, frequently after the development of complications [1,2]. We present a case of CS diagnosed in the setting of hypernatremia, hypokalemia, metabolic alkalosis and severe infection with the purpose of enforcing the importance of clinical suspicion and prompt diagnostic workup.

This article was previously accepted and presented as a poster at the European Congress of Internal Medicine (ECIM) 2022, on June 9-11, 2022 in Malaga, Spain.

## Case presentation

**Figure 1:**
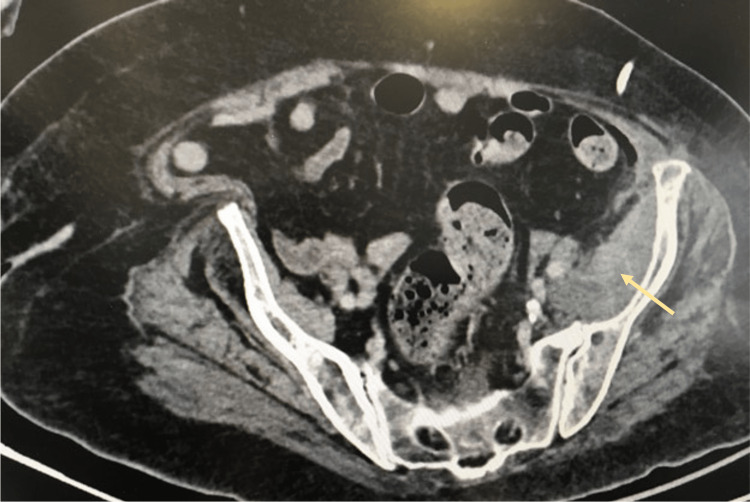
Left psoas abscess documented upon admission on computerized tomography.

An 82-year-old woman with obesity, long-stage (over 10 years) arterial hypertension and type 2 diabetes mellitus presented to the emergency department with fever and pain in the left hip. Because of incapacitating osteoarticular pain, she was being given intramuscular analgesia at the nursing care facility where she lived. Upon admission, her blood pressure was 120/80mmHg, temperature was 37ºC and she had pain with left hip mobilization, especially hip extension, but no local inflammatory signs. She presented a centripetal fat distribution and multiple non-traumatic bruises. She had neither violaceous striae nor buffalo hump. A computerized tomography scan showed an abscess (75x29x125 mm) along the left psoas muscle crossing the anterior surface of the hip joint. Figure 1 shows the mentioned abscess documented upon admission. Her blood analysis showed leukocytosis (25.580/µL) and a C-reactive protein level of 290 mg/L (reference range <3 mg/L), consistent with infection.

**Figure 2:**
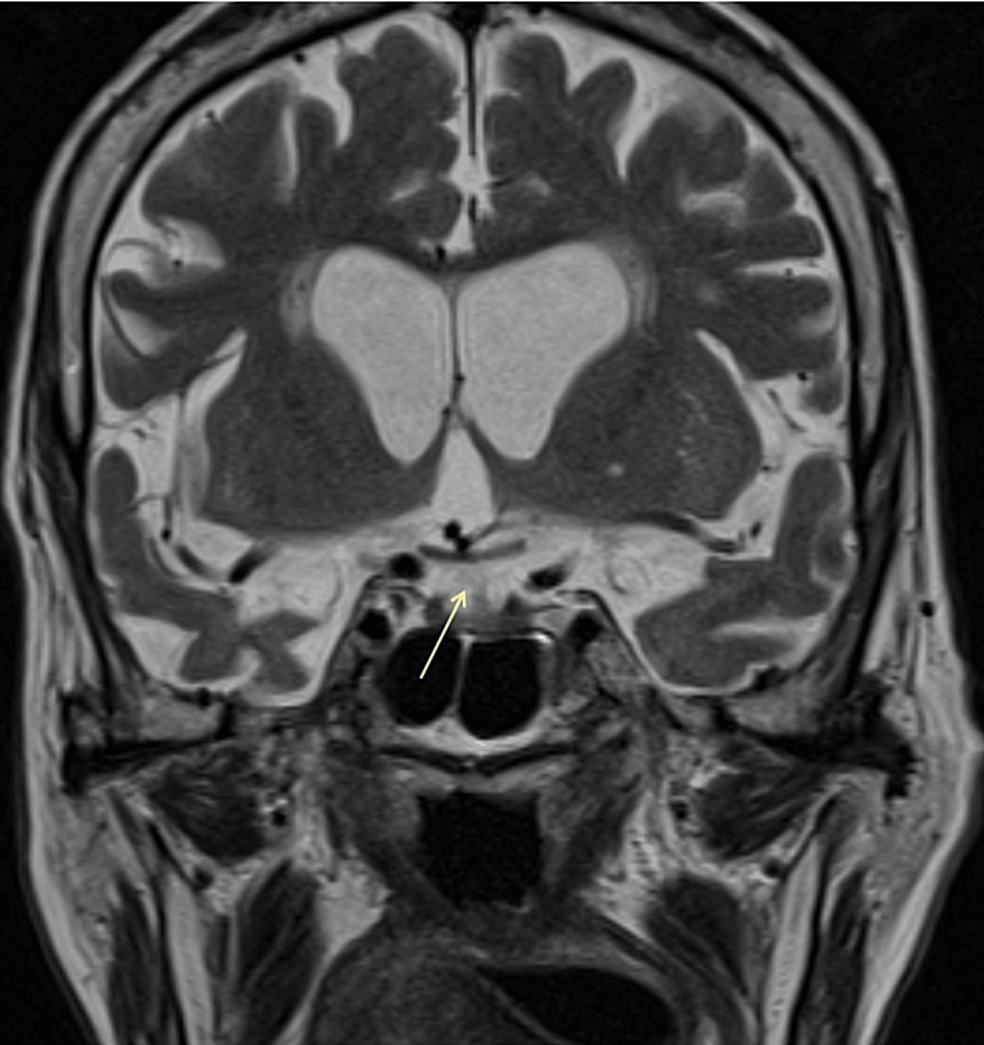
Right pituitary microadenoma on MRI. MRI - magnetic resonance imaging.

Broad-spectrum antimicrobial therapy was started with vancomycin and piperacillin-tazobactam - this choice took into account the fact that the patient resided in a nursing care facility and the absence of microbiologic data at this time. Antimicrobial therapy was switched to flucloxacillin when a methicillin-sensitive S. aureus was isolated in the blood cultures and the pus drained from the abscess. The echocardiogram ruled out infective endocarditis. The magnetic resonance imaging (MRI) showed two abscesses in the gluteus minimus and evolution to osteomyelitis of the hip joint. Concurrently, she presented hypokalemia resistant to supplementation, hypernatremia and metabolic alkalosis. These alterations made the team consider the hypothesis of hypercortisolism and exams were performed: overnight 1 mg dexamethasone suppression test was positive - cortisol 35.7 µg/dL (reference range 36-137 µg/day); midnight serum cortisol was elevated - 44.8 µg/dL (reference range 1.7-8.9 µg/dL) as were two 24-hour urinary free cortisol measurements - 609.1 µg/day and 1,636.6 µg/day (normal range: 36-137 µg/day); adrenocorticotropic hormone (ACTH) was 61.0 ng/L (reference range < 63.3 ng/L). The pituitary MRI showed a microadenoma. Figure 2 displays the microadenoma described in the MRI.

Despite percutaneous drainage and five weeks of directed antibiotics, surgical treatment was deemed necessary to control de infection. Treating hypercortisolism medically was intended, but the patient incurred a post-operatory fatal infectious complication. 

## Discussion

We present a case of ACTH-dependent CS without definitive etiology due to quick dismal evolution. The severity of the infection agrees with the hypercortisolism-induced immunosuppression. This electrolyte alterations combination - hypernatremia, hypokalemia and metabolic alkalosis - is mentioned in very few reports [5,6], but has a pathophysiological basis: hypercortisolism activates mineralocorticoid receptors in renal tubules, inducing an excessive mineralocorticoid activity, leading to increased sodium reabsorption, potassium excretion and increased bicarbonate reabsorption [5]. Therefore, this combination should bring hypercortisolism into consideration, especially when other causes are excluded.

It is recognized and accepted that hospitalization may induce a state of pseudo-CS ​​​​​[2]; however, the magnitude of cortisol values both in 24h urine (more than three times the upper reference range) and in the serum favor a real CS. ACTH-dependent CS is mainly due to a pituitary tumor. Less frequently, an extra-pituitary ACTH- or corticotropin-releasing hormone (CRH)-producing tumor can be the cause [1]. No clinical or biochemical features can differentiate between these two and and the gold standard is the catheterization of the inferior petrosal venous sinus. Our patient possibly had Cushing’s Disease, given its higher prevalence, the clinical signs of hypercortisolism, and the presence of a microadenoma [5]. However, she also had features more often present in ectopic syndromes such as marked hypokalemia and severe infection.

CS impairs immunity, both its innate and acquired components, and associates with an increased risk of severe infection and sepsis [​​​3,7].​​​​​​ The susceptibility to bacterial infections correlates with cortisol serum levels [7,8] and is more frequent in ectopic CS [7]. ​​Infection has been reported as the main cause of death within 90 days of CS diagnosis [9].​​​​​​

## Conclusions

The aggregation of signs, symptoms and comorbidities in our patient - easy bruising, hypokalemia, metabolic alkalosis, hypernatremia but also diabetes, hypertension and central obesity - raised suspicion of a hypercortisolism state. Despite this, the diagnosis was late, as a life-threatening complication was already present. In our case, we present a possible presentation of CS - hypokalemia, hypernatremia and metabolic alkalosis - and highlight the association of a late diagnosis, or long disease duration, with dismal outcomes.

## References

[1] Pappachan JM, Hariman C, Edavalath M, Waldron J, Hanna FW (2017). Cushing's syndrome: a practical approach to diagnosis and differential diagnoses. J Clin Pathol.

[2] Nieman LK, Biller BM, Findling JW, Newell-Price J, Savage MO, Stewart PM, Montori VM (2008). The diagnosis of Cushing's syndrome: an Endocrine Society Clinical Practice Guideline. J Clin Endocrinol Metab.

[3] Pivonello R, Isidori AM, De Martino MC, Newell-Price J, Biller BMK, Colao A (2016). Complications of Cushing’s syndrome: state of the art. Lancet Diabetes Endocrinol.

[4] Nieman LK (2018). Recent updates on the diagnosis and management of Cushing's syndrome. Endocrinol Metab (Seoul).

[5] Wassel E, Umeh CA, Giberson C (2021). Elevated adrenocorticotropic hormone, hypercortisolism, and marked hypernatremia. Cureus.

[6] Decaestecker K, Wijtvliet V, Coremans P, Van Doninck N (2019). ACTH-dependent hypercortisolism: always follow your nose. Endocrine Abstracts.

[7] Hasenmajer V, Sbardella E, Sciarra F, Minnetti M, Isidori AM, Venneri MA (2020). The immune system in Cushing's syndrome. Trends Endocrinol Metab.

[8] Pivonello R, De Martino MC, De Leo M, Simeoli C, Colao A (2017). Cushing's disease: the burden of illness. Endocrine.

[9] Valassi E, Tabarin A, Brue T (2019). High mortality within 90 days of diagnosis in patients with Cushing's syndrome: results from the ERCUSYN registry. Eur J Endocrinol.

